# Experience of Virtual Help in a Simulated BCI Stroke Rehabilitation Serious Game and How to Measure It

**DOI:** 10.3390/s25092742

**Published:** 2025-04-26

**Authors:** Bastian Ilsø Hougaard, Hendrik Knoche, Mathias Sand Kristensen, Mads Jochumsen

**Affiliations:** 1 Department of Architecture, Design and Media Technology, Aalborg University, 9000 Aalborg, Denmark; hk@create.aau.dk (H.K.);; 2 Department of Health Science and Technology, Aalborg University, 9220 Aalborg, Denmark

**Keywords:** serious games, stroke rehabilitation, brain-computer interface, frustration, agency, perceived control, input assistance, assistive technology

## Abstract

Designers of digital rehabilitation experiences can accommodate error-prone input devices like brain–computer interfaces (BCIs) by incorporating virtual help mechanisms to adjust the difficulty, but it is unclear on what grounds users are willing to accept such help. To study users’ experience of virtual help mechanisms, we used three help mechanisms in a blink-controlled game simulating a BCI-based stroke rehabilitation exercise. A mixed-method, simulated BCI study was used to evaluate game help by 19 stroke patients who rated their frustration and perceived control when experiencing moderately high input recognition. None of the help mechanisms affected ratings of frustration, which were low throughout the study, but two mechanisms affected patients’ perceived control ratings positively and negatively. Patient ratings were best explained by the amount of positive feedback, including game help, which increased perceived control ratings by 8% and decreased frustration ratings by 3%. The qualitative analysis revealed appeal, interference, self-blame, and prominence as deciding experiential factors of help, but it was unclear how they affected frustration and perceived control ratings. Building upon the results, we redesigned and tested self-reported measures of help quantity, help appeal, irritation, and pacing with game-savvy adults in a follow-up study using the same game. Help quantity appeared larger when game help shielded players from negative feedback, but this did not necessarily appeal to them. Future studies should validate or control for the constructs of perceived help quantity and appeal.

## 1. Introduction

Strokes are the third leading cause of death, and disability is set to worsen in aging populations [1]. Most stroke survivors require extensive physical exercises to restore mobility [2]. To do this, a novel approach uses a brain–computer interface (BCI) to improve upper limb mobility [3], but its training requires many repetitions, and the BCI suffers from low input recognition [4].

Games are more engaging than standard exercises and can motivate patients to perform many such repetitions [5,6], but the level of difficulty of the game should be adequate to not frustrate patients unnecessarily. Frustration can create a vicious cycle by reducing the recognition of the BCI input, increasing frustration [7,8]. To counter low input recognition, the BCI rehabilitation engineering community has focused on improving hardware and classification accuracy, while the human–computer interaction (HCI) community suggested focusing on game design to solve inaccuracy shortcomings [9]. To achieve optimal game experience, dynamic difficulty adjustment (DDA) techniques generally tweak games to match players’ abilities [10] based on performance, affect, or manual interventions [11]. Similarly and specifically to address low input recognition, Rossau et al. proposed performance accommodation mechanisms (PAMs) to provide players explicit help, designed to increase perceived control and avoid frustration [12]. However, no study has investigated how patients experience such explicit help when faced with imperfect input recognition and how this should be measured.

In our first study, naive patients experienced explicit help when controlling an experimental protocol game featuring a task with multiple action attempts and artificially pre-defined input recognition rates. The second study empirically builds on the first to deepen the methodological approach with a proxy group, measuring the perceived amount and appeal of the help.

Our contribution uncovers stroke patients’ experience of help to establish four relevant experiential factors (help quantity, appeal, pacing, and irritation). When controlling for these experiential factors, non-stroke participants differentiated the experience of provided help by help quantity and appeal and not by their perceived control or frustration.

### Paper Structure

- The background (Section 2) outlines how BCIs and games are used in stroke rehabilitation, motivating the need for game help.
- Study 1 (Section 3) describes the procedure and results of a study game that helps 19 stroke patients.
- Study 2 (Section 4) presents a follow-up study with 20 healthy subjects based on findings from Study 1 and explores 20 additional experiential measures of game help.
- The discussion (Section 5) reflects on the protocol design evolution, clinical implications, and how the work paves the way for future studies of game help.

## 2. Background

In stroke rehabilitation, patients exercise daily to recover motor function, following treatment individualized to their disabilities [2]. For patients to relearn their lost motor skills, rehabilitation exercises require many repetitions of demanding movements, which makes motivation critical [13]. For patients with acquired brain injury, low motivation is associated with low adherence and poor rehabilitation outcomes [14]. Reviews of patient adherence to therapy in developed countries have shown that adherence only averages 50% [15]. Some of the key factors to improve adherence among older adults in traditional physical therapy involve fostering high self-efficacy, high self-motivation, and feeling that the treatment is helping [16]. Patients’ inclination to adhere to treatment may improve with technological intervention; for example, in a long-term study comparing technological and analog interventions, patients felt that the technological intervention contributed more to the rehabilitation of their upper-limb function than patients using an analog counterpart [17].

### 2.1. Rehabilitation with Brain–Computer Interfaces

BCIs provide a potential form of rehabilitation, which has shown positive clinical outcomes in controlled research studies and laboratory settings. Rehabilitation with BCI involves recording the patient’s electrical brain activity while the patient repeatedly imagines movements of their paralyzed limb, known as motor imagery (MI). While the patient performs MI, the BCI extracts the patient’s movement intention and triggers functional electrical stimulation of the muscles in the affected limb to provide somatosensory feedback that is congruent with the movement intention, which promotes functional recovery [18]. Depending on various factors such as volume conduction, attention, fatigue, and brain injury (such as stroke), the performance of movement intention detection of BCIs is limited to a true positive rate of approximately 70–90% and, in some cases, lower [19,20,21]. A true positive rate in this range is good enough for neurorehabilitation from a clinical point of view [20,22]. However, the difficulty in obtaining consistently higher true positive rates combined with the patients’ impairments restricts their access to the ground truth; patients do not know if they are correctly imagining movements or responsible for triggering the BCI, which reduces their experience of control.

A BCI is an imprecise input device in which users’ interaction may not always lead to a certain outcome due to the inherent variability of brain activity. Unlike a mere button press, an imprecise sensor may (1) fail to register user input (false negative) or (2) register user input when, in reality, the user had no input intention (false positive). Such error-prone interaction may eventually lead to a perceived loss of control and frustration for users. Users experience loss of control as a pre-emotional state when events impede them from achieving their goals, creating immediate frustration and a long-term impact on their mood [23]. For BCI, this frustration leads to lower input recognition [24]. In a study of BCI error rates and frustration, BCI error rates increased self-reported frustration, suggesting a vicious cycle [25].

Avoiding frustration with error-prone input devices is difficult because people expect technology to work as a consequence of external agency (feeling in control over external objects or the environment), which is a natural extension of body agency learned from experience [26]. When people encounter technologies with which their actions do not cause external effects in the traditional cause–effect sense of HCI, they feel a loss of control [27]. Examples of such imprecise input technologies include action augmentation systems [27], such as BCIs, prosthetics, voice-based interfaces, and gesture-based interfaces. BCIs’ imprecise input is one of the technological challenges that hinder their adoption in rehabilitation [28].

### 2.2. Games for Rehabilitation

Video games have the potential to increase patient motivation in rehabilitation [29,30,31,32,33] as complementary activities to existing rehabilitation activities and therapy [34,35,36]. For example, clinicians suggested in focus group interviews that technological interventions could supplement their facilitation by continuously providing information about exercise completion, speed, and quality [37]. Exergames represent a sub-genre of video games which combine rule-based play with physical movements to create sustained exercise [38]. They are used in rehabilitation as rehabilitation games to train a wide range of physical deficits from muscle weakness (e.g., hemiparesis) to complete limb paralysis (e.g., hemiplegia), and exergames border cognitive rehabilitation at this extreme. Rehabilitation games serve as extrinsic motivation through rewards and intrinsic motivation, providing engaging play and challenge [14]. Both types of motivation play an important role in the success of rehabilitation [14]. In a meta-analysis, extrinsic motivators made people train more (performance quantity), whereas intrinsic motivators improved how well they trained (performance quality) [39].

Game researchers have sought to understand how to design exergames tailored to specific rehabilitation exercises to provide a large range of difficulty adjustments [40]. For example, researchers have employed knowledge-driven design approaches to tailor exergames to exercise elbow flexion and extension in VR [41] and improve cognitive impairments like amnesia [42]. For exercises with BCI, games need additional tailoring to accommodate the BCI’s imprecise detection of MI and avoid an erroneous and frustrating experience that may hinder long-term use [28].

### 2.3. Game Help: Improving Perceived Control and Frustration

To address frustration in games, game designers can incorporate dynamic difficulty adjustment (DDA), which modulates gameplay to match players’ abilities [10]. Researchers have studied the effectiveness of different adjustment strategies, including how manual adjustment compares to adjustment based on players’ performance or through player profiling (player behavior models) [43]. Adjustment strategies can be informed automatically by affective feedback loops that recognize users’ psychological state continuously to inform when to trigger task adjustments [44]. Some players find externally controlled task manipulation confusing due to misinterpreted feedback, incorrect attribution (who caused what), or conflicting expectations [45].

Other studies have shown that adaptation mechanisms that tailor experiences to players can improve the resulting player experience [43]. For example, manual and performance-based adaption of an arm rehabilitation exercise led to higher motivation and exercise intensity [11].

Once the system recognizes a situation in which difficulty adjustment is needed, the system triggers a designated adjustment mechanism, of which different approaches exist. DDA can be designed to lower the challenge of the game by adjusting parameters that make the game easier or harder. For example, Goršič et al. altered ball speed and paddle size in a pinball game, effectively making the game more or less challenging [11]. Other forms of DDA include time manipulation to alter game difficulty [46], reducing the number of input controls required (‘one-switch’) or providing extra input (known as ‘input automation’) [47]. Input automation maintains the same game challenge but provides help implicitly on the input side (as an additional input to the BCI input). For example, input fabrication induces an artificial signal that results in users receiving more positive outcomes and a heightened sense of control [48]. Players’ experience of control is also referred to as perceived control [49] or sense of agency [27]. Input fabrication relies on concealment to create an illusion of control from receiving more positive feedback, initially observed in causation studies within experimental psychology [50].

### 2.4. Performance Accommodation Mechanisms as Game Help

Contrary to input automation, performance accommodation mechanisms (PAMs) provide explicit help by creating narratives in relation to in-game events [12]. PAMs aim to boost successful outcomes or reduce the impact of negative outcomes. These mechanisms exploit how game environments can manipulate outcomes deliberately to make imprecise input acceptable [51]. PAMs are a set of conceptual help mechanisms that describe ways to augment player input and enhance players’ experience in poor performance scenarios. A study exploring three PAMs with healthy subjects using an online BCI [52] found that help mechanisms increased perceived control for poor BCI users and decreased perceived control for well-performing BCI users. However, little is known about patients’ experience of PAMs to accommodate low input recognition.

Study 1 investigated how patients experienced help from three types of PAMs compared to a control condition. *Augmented success* doubled the meaningful outcome of player input; *input override* made a system-controlled character take over the player’s task and *mitigated failure*, preventing any negative outcomes from happening as a result of lack of player input [12].

### 2.5. Methodology: Simulating the BCI to Study Game Help

To measure perceived control and associated frustration in BCI and DDA contexts, studies must account for both within- and between-participant variation in ability and input recognition. BCI recognition rates can vary hugely across participants, even in laboratory settings, due to inter-individual differences, a low signal-to-noise ratio, and non-stationarity of the electrical brain activity [53]. For example, in previous studies of help in laboratory settings, recognition rates varied from 11% to 92% [52] and 11% to 88% [54] in lab settings.

Since researchers cannot experimentally manipulate BCI input success rates, some use a reliable replacement input method instead to create artificially predefined classification accuracies [25,49]. Study designs that use predefined classification accuracies rely commonly either on simulation [55,56] or Wizard of Oz approaches [44,57]. Simulation methods are employed in non-BCI studies of dynamic difficulty adjustment scenarios, like affective computing, to control the classification accuracy in relationship to users’ play experience [44,56]. For example, in a Wizard of Oz study by Novak et al., participants were led to believe an algorithm controlled the task difficulty via sensors tracking users’ psychological state, while it was controlled by study facilitators at artificially predefined rates [44]. Rather than using a human to control outcomes (Wizard of Oz), simulation studies can employ a different input device with a high recognition rate [49,51]. The recognition rate of a sensor quantifies its ability to pick up user input, where picking up user input 8 out of 10 times corresponds to a recognition rate of 80%. By using a high-recognition sensor as a simulated BCI signal, researchers can then artificially introduce inaccuracy in a controlled manner. This provides access to a ground truth of what happened exactly when which allows for understanding the causal relationship between the user’s input and the system’s feedback at predefined recognition rates. Simulating the BCI effectively separates the BCI’s recognition from user interactions and provides increased experimental control.

#### Blinking: A Proxy to Simulate Motor Imagery BCI

To increase internal validity in BCI simulation, studies have simulated properties of BCI interactions to varying degrees: (1) leading users to believe they operated a BCI when wearing a non-operational BCI headset while interacting with applications designed for BCI input [25,56], and (2) incorporating BCI characteristics into a different input modality, like pressing buttons [49] or blinking [48]. Blinking offers valuable properties that make it a viable proxy for motor imagery BCI interactions because both are not commonly used as an input device, creating an unfamiliar experience. Performing motor imagery attempts with the BCI creates uncertainty about how correctly they were executed, and people can likewise be uncertain about their blink attempts when instructed to blink in certain ways (e.g., slow/fast, hard/soft, long/short, and determined/casual), which carry enough ambiguity. Unlike BCI motor imagery attempts, blinks can be observed. These properties make blinking suitable for simulating imprecise input devices while possessing near-perfect input recognition when in eye-tracking range. Blinking was previously used as a simulation method in a BCI context and showed agreement between simulated and true online BCI control [54].

Taking a simulated BCI approach similar to Hougaard et al. [48], we adopted the procedure and the game apparatus of an online BCI study [52] and used a simulated BCI to test three PAMs with stroke patients. In other words, we switched the BCI recognition with blink recognition, and the software simulated the behavior of a BCI, providing control with low input recognition. Pilot studies with four healthy subjects were conducted on the three virtual help mechanisms to verify the test procedure and the BCI simulation before running the study with stroke patients. We applied the results of the stroke patient study (Study 1) to explore new self-report measures of help with game design students (Study 2).

### 2.6. Summary

PAMs offer strategies to provide explicit help when users face low input recognition from BCIs, but little is known about how stroke patients experience such help mechanisms. Our study’s goal was thus to capture how stroke patients respond to game help in terms of perceived control (their sense of being in control of events happening in the game) and frustration when engaged in rehabilitation exercise controlled by an imprecise input method (blinking). What are the main factors that affect patients’ frustration levels in the design of help? What kind of help do patients think of as appropriate, if any? Investigating patients’ agency and frustration could uncover what underlies patients’ willingness to accept or reject different types of help, all of which are crucial information for designers. Based on the observations and lessons learned from the stroke patient study, we then conducted a follow-up exploration, in which we derived novel ways to measure what underlies user responses to help in rehabilitation games.

## 3. Study: Stroke Patients’ Experience of Game Help

We designed a mixed-method field study in a within-subject experiment conducted in a rehabilitation center in Denmark, where patients performed a blinking task during four variations of a digital fishing game. At the time of running the study, the institutional ethical review board, upon request, did not deem the study to require in-depth ethical approval (journal inquiry 2021-000438 confirms this affirmation).

### 3.1. Participants

Nineteen sub-acute stroke patients between 23–78 years old (M = 61) participated in the study. A trained physiotherapist screened them for their cognitive abilities and based on a description we provided of the study. All stroke patients had to have the cognitive ability to meaningfully answer a questionnaire. This excluded patients with signs of aphasia (an inability to speak and comprehend speech), blindness, and patients who were bedridden or had neurodegenerative diseases. The clinical team at the rehabilitation centers otherwise had the discretion to invite patients they saw fit, and we monitored if patients showed any signs of challenge during their participation. Stroke patient lesions were not CT scanned prior to the experiment, and there were no requirements regarding their motoric ability since the only required interaction was blinking. This meant that patients with hemiparesis and hemiplegia were not excluded.

### 3.2. Procedure

**Figure 1:**
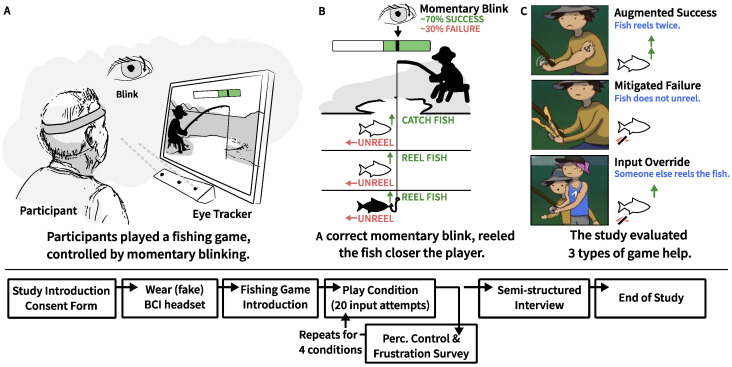
In the simulated BCI study, participants wore fake BCI headsets and controlled a fishing game by blinking (**A**), which reeled a fish when successfully recognized. If unsuccessful, the fish tried to escape by moving horizontally away (**B**). We evaluated three help mechanisms: augmented success strengthened the fisherman, mitigated failure clamped the fishing rod, and input override introduced a person to help (**C**). The bottom flowchart shows the study’s progress.

The experiment lasted 30 min per participant and consisted of an introduction (5 min), playing the fishing game (20 min), and a semi-structured interview (5 min). To play the fishing game, we employed a non-intrusive, imprecise control method based on blinking adopted from Hougaard et al. [48], which was a novel and unusual input method for all participants. During the introduction, the facilitator explained the game from an image shown on a printout, which can be found in Supplementary Material S1. Participants wore a BCI headband (a single-electrode Myndband by MyndPlay Ltd in London, UK) and were instructed to play a game, which reacted to eye blinks picked up from the headband. However, in reality, the BCI headband did not record participants’ eye blinks, and no BCI was actively used to interact during the experiment. Instead, an eye tracker (EyeX manufactured by Tobii in Stockholm, Sweden) mounted on the game laptop continuously recorded blinks with high precision, as illustrated in Figure 1 (left). A blink consisted of momentarily closing and opening the eyes. The system waited until participants opened their eyes and either responded to the blink or not. The response depended on a predefined schedule that emulated input imprecision.

Participants had to rate their experience after each of the four conditions, in which they played a fishing game designed by Jochumsen et al. [52]. In the first (control) condition, the participant played with no virtual help, i.e., only their own blinks could reel up fish. In the three other conditions, the participant received virtual help from the system (Figure 1, right). The participants had 20 attempts per condition to catch as many fish as they could. After each condition, participants rated their frustration and perceived control of the game on seven-point Likert scale items (see Supplementary Material S1) based on the frustration scale from Evain et al. [25] and perceived control from Greville and Buehner [50].

After all four conditions, the facilitator interviewed the participants. We designed a semi-structured interview to understand the participants’ experience and supplement quantitative data (the interview guide is available in the *Debrief* section of Supplementary Material S1). Participants first had to pick what they thought to be the hardest and easiest condition. Then, participants elaborated on (1) their prior expectations for the experiment, (2) their thoughts about game help, (3) the reasons for their ratings, and (4) whether they experienced mental fatigue.

### 3.3. Apparatus: Fishing Game

**Figure 2:**
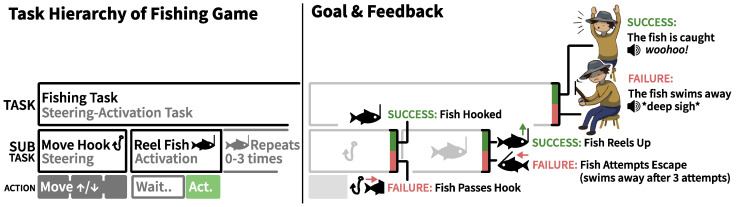
(**Left**): The game’s task hierarchy consisted of one main fishing task and two subtasks: moving the hook and reeling in the fish. Underlying core tasks are denoted in gray. (**Right**): Feedback is presented whenever a player fails or succeeds any task or subtask.

The study investigated three types of virtual help situated within a fishing-themed rehabilitation game in which players controlled a fisherman whose goal was to catch fish by reeling them up from a lake (an *obtainment* goal in Cardona-Rivera and Rogelio E.’s game goal typology [58]). The study employed this fishing game as it was designed and developed for motor imagery-based BCI rehabilitation [52]. To fit stroke patients, the game allowed players to interact at their own leisure in a peaceful setting instead of demanding high pace or startling events. Following a task-analytical approach [59], we chart the game’s designed interactions and feedback in Figure 2. The game’s task consisted of two subtasks: (1) moving a hook up or down towards the fish swimming in horizontal lanes using the laptop’s arrow keys and (2) reeling a hooked fish upwards to catch it using imprecise input. In BCI motor imagery rehabilitation, patients wearing BCI sensors must repeatedly attempt motor imagery input during a trial period, followed by a rest period [60]. The fishing game facilitated this pattern during its fish-reeling subtask with a white and green bar representing the rest and input (trial) periods (as shown in Figure 1, middle). When a fish was hooked, a black cursor began moving from left to right within the bar displayed above the fisherman. To reel, players had to blink quickly once during the trial period, i.e., from when the black cursor entered the green area before reaching its end. When players were (somehow) successful at reeling, the fish would be reeled up, getting closer to the fisherman (Figure 1, middle). When the fish was in the top-most lane, a successful blink removed the fish from the lake and resulted in a catch, completing the fishing task.

However, players’ control was designed to be imprecise; players experienced that the system would not always respond to their blinks. Players were unaware of the exact input imprecision introduced by the system, which was set to recognize blinks only during 70% of the trial periods. After each failed trial period, the fish moved towards escaping by swimming away (by one column). After three failed reel periods, the fish escaped.

#### Fishing Game Help

**Figure 3:**
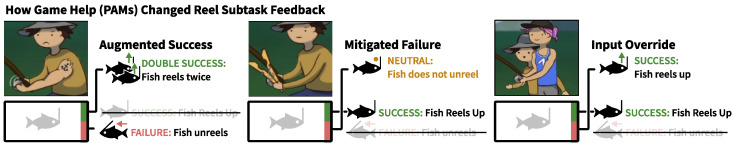
When the system activated game help, reel subtask feedback was overwritten with enhanced outcomes that improved the player’s chances to catch the fish.

The game incorporated three types of help (Figure 3), designed to behave distinctly from each other to allow for discussions during the interview. *Augmented success* boosted successful blinks (reels), i.e., the fisherman temporarily got stronger, reeling fish through two lanes instead of one. *Mitigated failure* helped players at the end of a failed reel period. The fisherman clamped his fishing rod to prevent the fish from moving away in one column. *Input override* also helped players during such failed reel periods, i.e., a woman ran over to the fisherman and reeled up the fish for him. The game help conditions each provided up to 30% of extra help. All conditions ensured that participants experienced system imprecision, i.e., failed reel trials. An eye tracker captured blinks with high recognition rates when participants were within range. To simulate imprecise input, the game artificially lowered success rates by presenting negative feedback (failed attempts) to maintain a 70% success rate. All participants, therefore, experienced 70% precise control of reeling or less during the experiment. In the reference condition, participants, therefore, received up to 70% positive feedback (successful reels) and at least 30% negative feedback (failed reels).

### 3.4. Data Analysis

**Figure 4:**
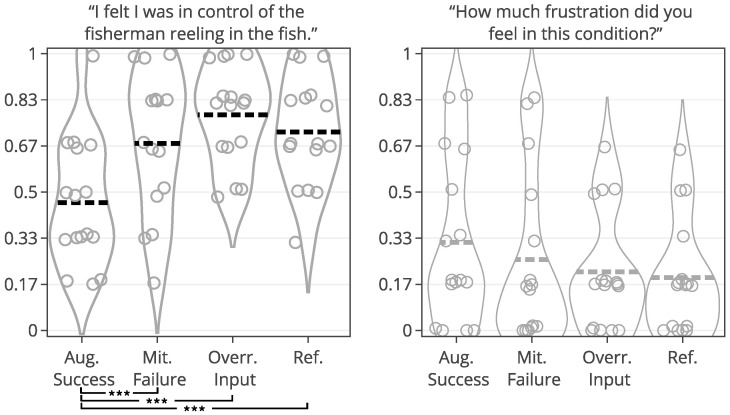
Participant ratings (circles) and mean (dashed line) of perceived control (**left**) and frustration (**right**), normalized with 2% jitter. Significant differences between conditions (***) are marked underneath in black.

The participant ratings and quantitative game performance data are explained in Table 1. The means of each measure per condition are available in Table 2. Likert scale measurements were visually analyzed as violin plots overlaid by data points corresponding to participant scores. The violin plots’ shape allowed us to visually understand and compare rating distributions and highlighted rating majorities. To mitigate overplotting, we normalized the data points from 1–7 to 0–1 and jittered them by 2% in Figure 4. However, subsequent analysis treated Likert scale measurements exclusively as ordinal measures. We marked significant differences under the violin plot of each condition, if any. The collected data were analyzed in R studio using the ordinal package [61].

In addition to these measures, the influence of participant fatigue, gender, and condition order was tested. The included game measures were, by design, not fixed in the experiment to maintain participants’ agency and stake in outcomes and prevent outcome prediction between conditions. Standard deviation and intraclass correlation (ICC) were calculated for each self-reported measure to estimate the consistency with which the participants rated their perceived control and frustration. We constructed cumulative link mixed models and used the models’ Akaike information criterion (AIC) and maximum likelihood (ML) to understand how help and game feedback affected participants’ ratings. The models were fitted with the Laplace approximation, and the participants were considered random intercepts to account for by-subject baseline rating differences. We tested help and feedback variables as fixed effects using likelihood ratio tests with a *p*-value threshold of 0.05. We used open coding analysis [62] of the qualitative data to identify themes in participant answers and observations. Representative quotes of the found themes were translated from Danish and included in our results. While most stroke patients were capable of performing the blinking task, they could occasionally lean outside the recognition area or forget to blink as instructed. After collection, data were normalized, blink recognition rates were calculated, and abnormal recognition rates were further examined using video analysis. Due to technical difficulties, three participants (7, 11, and 15) had no blink recognition at all, and their data were thus excluded from subsequent quantitative analysis.

### 3.5. Results

Likert scale ratings and player performance means are available in Table 2. For each self-report measure, we tested models made from the game measures listed in Table 1, which yielded nine significant models for perceived control and six significant models for frustration, listed in Table 3. Six participants reported experiencing mental fatigue from playing (P2, P3, P5, P6, P8, and P9) after the experiment was over. We constructed models with fatigue, gender, and condition order as random effects but did not identify significant relationships between participants’ ratings of perceived control and frustration. The blink conversion rates in Table 2 were low, indicating that participants blinked more than once per reeling attempt and that the majority of their blink attempts had no effect on the game. On average, patients performed two blinks for every reeling attempt and blinked on average 42 times (SD = 16). We observed high engagement in catching fish among patients, and the majority (11/19) received the game well. The remaining eight participants expressed mixed opinions of the game (repetitive, silly, and overwhelming).

#### 3.5.1. Perceived Control

Overall, the augmented success condition scored lowest in perceived control (M = 3.75/7) and highest in frustration scores (M = 2.88/7), as also depicted by the violin plots in Figure 4, which visualize the means normalized from 0–1 per condition. Our model analysis in Table 3 showed that when patients’ rated perceived control, their ratings were best explained by a feedback-based model of how many fish participants had reeled (AIC = 193.68, ML = −89.84, and *p* < 0.001), listed as “(1|Participant) + Fish Reel” in Table 3. In this feedback-based model, reeling fish affected participant ratings positively by 0.61 (8%) (each fish reeled increased perceived control by 0.61). The model estimated medium variance between participant ratings of perceived control (SD = 2.31). Augmented success incurred the lowest amount of positive feedback (30%), which explains participants’ low perceived control ratings (see Figure 4, leftmost violin plot). We created a condition-based model “(1|Participant) + Condition”, which used the experimental conditions (three types of help and reference condition) to predict perceived control ratings. The model estimated positive influence from the input override condition (0.76) and negative influence from augmented success (−2.69) and mitigated failure (−0.30) on users’ perceived control. Perceived control ratings of augmented success were significantly lower than those for mitigated failure (*p* = 0.003), input override (*p* < 0.001), and the reference condition (*p* < 0.001) (see Figure 4). Other mean comparisons between help conditions did not differ significantly. The condition-based model yielded a worse explanation of ratings with lower AIC and ML (AIC = 209, ML = −96, and *p* < 0.001) compared to the feedback-based models like fish reel (AIC = 193, ML = −90, and *p* < 0.001). No significant models emerged from combining help conditions and positive feedback, which suggested a high model overlap and co-variance between the help conditions and positive feedback. This model overlaps between positive feedback, and help models were expected, considering that adding help introduced more positive feedback. Augmented success had the lowest blink conversion rate and positive feedback, averaging lower than the reference condition, which was unexpected. Due to the design of the augmented success mechanism, the condition yielded the lowest blink conversion rate and provided the least help. Users could only receive augmented success within the second and third lanes, where the fish had to reel up double the distance.

Participants expressed different opinions about the game help. In augmented success and mitigated failure conditions, patients expressed difficulty perceiving the added help (P10, P16, and P17). P10 commented on the mitigated failure condition’s prominence: “*I didn’t really notice the clamp, but I think I did really well*”. In the augmented success condition, participants’ high loss of fish overshadowed the help they received (P1, P5, P10, P16, P17, P18, and P19). “*I lost way more fish this time, it felt like it was hard to control. I did my best…*” (P18). In the input override condition, some patients (P6, P9, P16, and P10) appreciated the help: *“It was nice that she helped. She took the fish up a notch at times, when I couldn’t”* (P10). Other patients disliked the interference (P8, P18, P7, P1, and P17), e.g., P8 complained *“She shouldn’t interfere in the game. I’m catching many fish anyway [without her help]”*.

#### 3.5.2. Frustration

Patients reported low frustration in all experimental conditions despite losing fish, which is also evident from the frustration means in Figure 4’s right-most violin plot (M = 2.44 and SD = 1.55). No condition-based models predicting frustration were significant, suggesting that patients’ frustration ratings were not influenced by the type of help they received. Our feedback-based models in Table 3 proved significant and were equally good at explaining patients’ ratings of frustration. One model estimated that reeling fish decreased patient frustration by −0.24 (AIC = 186, ML = −87, and *p* = 0.011). Another model estimated that losing fish increased patient frustration by 0.37 (AIC = 187, ML = −87, and *p* = 0.015). In total, 7 out of 19 patients expressed frustration in relation to playing the game or during interview debriefs. *“It was specially annoying when the fish was all the way to the right [close to losing it], and didn’t want to come up”* (P15). Four patients, on the other hand, explained that they did not feel the game affected them in a frustrating way (P2, P4, P7, and P11). *“Fishing [and losing fish in a game] is not something that annoys me”* (P2). Two patients (P5 and P6) explained that their frustration came from their inability to play well and from the game itself. When rating condition difficulty, 7/16 participants rated augmented success hardest and 10/16 participants rated the reference condition easiest.

### 3.6. Study Discussion

In this study, 19 stroke patients evaluated four variations of a video game, in which we provided three different help mechanisms which contrasted each other. The stroke patients shared their viewpoints and rated the games in terms of their frustration and perceived control.

#### 3.6.1. Help Preferences and Perceived Control

Mean Likert scale ratings of perceived control in Figure 4 indicated that stroke patients felt most in control when helped by input override and least by augmented success. The help type and positive feedback models overlapped, and the feedback model explained more variance than the help type. The model overlap is expected since, by design, each help type changed the overall quantity of positive feedback. The model overlap suggested that positive feedback quantity (reeling and catching fish) was more important than the narrative frame of help (becoming stronger, external help, and avoiding failure). Most patients felt higher agency from the 27% extra positive feedback they received from the input override, even if it meant accepting that a virtual character took control over their fishing rod. In contrast, patients felt less in control when helped by augmented success due to the 28% average reduction in positive feedback frequency provided in the condition despite its double reels. These observations in the quantitative data were backed up by patients’ responses about doing well that related to the task outcomes: catching and losing fish. Even though help was communicated as being beneficial to their reeling subtask (double reels), patients’ experience prioritized the lower quantity of positive task feedback from catching fish. Results from our previous study [52] of augmented success, input override, and mitigated failure are not comparable due to differences in target group (patients vs. healthy subjects), control method (blinking vs. real BCI), and the continuum of control (fixed base amount vs. varied amount). We observed a larger willingness among patients to accept help from input override than in our previous study, where participants’ preferred input override the least over any other condition regardless of the control amount.

#### 3.6.2. Help Preferences and Frustration

None of the virtual help significantly affected patients’ frustration, who rated it low for all conditions. Yet, patients blinked many times during trials selected for failure and clearly indicated frustration during a playthrough, for example, when losing many fish in the augmented success condition. With our baseline at 70% classification accuracy, we did not find clear signs of near-inverse correlations between frustration and perceived control, as found in previous study contexts [48,52]. When asked about their frustration, four patients explained that events of the game itself could not frustrate them, two patients blamed their own performance, while the remainder remarked varying degrees of frustration in relation to blinking (action), receiving help (subtask), and losing fish (task)—all sensible reasons which are understandably difficult to capture when mixed in a single rating. We calculated the ICC for self-report measures in Table 2 to assess the reliability of ratings, which showed low agreement among participants. We know from previous game rehabilitation studies with stroke patients, e.g., [63], and from motor impairment research [64], that such inconsistencies in quantified measurements are natural to find in stroke contexts due to the individual health conditions of the patients directly affecting individual performance and strategy differences. Inconsistencies can also become difficult to identify, particularly in game studies, due to the natural and unpredictable variation in game outcomes [65].

#### 3.6.3. Blink Sensor Reliability with Stroke Patients

The mean values in Table 2 indicate that the eye tracker reliably captured blinks in most stroke patients. We measured high blink recognition (sensor reliability) for most patients throughout the experiment with a median of 100% (M = 88%, SD = 0.21). The recognition rates from the eye tracker were better than observed in previous BCI studies with healthy subjects, where conversion rates spanned 10–90% [52,54]. The high reliability of the blink sensor, together with video recordings, allowed measuring the true recognition rate by comparing recognized input to the true total number of input attempts, which is not feasible with real BCI input and, therefore, not measurable in previous BCI-based studies. The recognition rates from the blink sensor had more variation than anticipated, which prompted an inspection of the true recognition rate for irregularities. Video footage indicated that patients at times leaned out of the eye tracker’s range and at times were not blinking as per instruction, perhaps forgetfully. These behaviors were partially unanticipated side-effects of patients’ stroke condition since a common side-effect in stroke is motor neglect and hemiplegia, where some people experience weak control of one side of the body, which can hinder their ability to sit up straight, and alter the subjective perception of what is vertical, which may distort further when patients become fatigued [66]. Future simulation studies utilizing blinking should consider how to mitigate the effects of leaning through, e.g., verbal reminders, head support, or gentle correction from a healthcare professional.

#### 3.6.4. Study Procedure with Stroke Patients

A number of participants reported scores that misrepresented what they expressed in the interviews. For example, when subjects rated frustration, augmented success with its low positive feedback rate was often rated equivalent to other conditions in terms of frustration, despite the patients weighing positive feedback highly in their judgement of help. Mismatches between quantitative results and participant qualitative accounts are not uncommon in human experiments. In our study, these mismatches stemmed from three challenges: (1) missing the frame of evaluation (e.g., P2, P4, P7, and P11 referring to the game overall not frustrating them), (2) temporal granularity (patients rated frustration, and their oral responses hint that further detail is available), and (3) dimension (rating depicted a one-dimensional scale, but patients’ responses suggested multiple possible extremes/sub-dimensions). To this date, we are unaware of suggestions about how to alleviate such inconsistencies except through extensive pilot studies with stroke patients. However, these are logistically difficult, expensive, and ethically dubious. We took the following preventive measures to minimize risk: (1) we used a simulated input method, which we had already successfully used with stroke patients in a previous study [54]. (2) We internally tested the experimental protocol and conditions with healthy subjects during their development. (3) We were prepared to intervene when patients at various points made errors in terms of handling input devices (arrow keys and blinking), forgot instructions or how to interact, misinterpreted questions and scales, or took liberties with following instructions.

#### 3.6.5. Augmented Success Design Questions

Augmented success was rated the hardest by seven participants. Many participants in the augmented success condition had fewer fish caught, less positive feedback, and more fish lost than in other help conditions and the reference condition. The lower performance in augmented success is partially by design since augmented success boosts the successful reeling of fish but does not prevent losing fish (see Figure 3). However, participants’ performance were lower than anticipated. Ground truth inspection of the simulated blinking signal revealed the main cause: augmented success only doubled positive feedback if the fish position allowed for reeling double distance. We took this measure to ensure that augmented success always had a consistent doubling effect whenever the mechanism was activated. On reflection, the chosen activation design did not do the help mechanism justice because augmented success would reduce the overall positive feedback rate (fish reel and fish caught) when not activated. The fishing game, therefore, ignored more successful input when using augmented success due to its consistent doubling effect compared to the other help mechanisms. Two solutions could ensure consistent activation rates: (1) minimizing situations in which fish cannot be reeled double, and (2) letting augmented success activate in situations where it effectively only reels fish up one lane.

#### 3.6.6. Limitations

Our study intended to evaluate the user experience of distinct types of explicit game assistance in lab settings to avoid frustration in people with diminished control over their lives and bodies. The study did not intend to understand whether the serious game we used or its type was a good fit for rehabilitation.

The sample size used for the study lies in the same range as similar studies in this field [41,67]. However, we acknowledge that the sample size chosen for the study may not have been large enough to support the application of inferential statistics.

Limitations regarding the external validity of our results study include the fixed success rates, occurrence patterns of help, novelty effects, game type and genre, the test population, the choice of simulated BCI input, and methodological measurement artifacts.

Players might react differently to the provided help types when faced with markedly lower, higher, or varying success rates. However, the chosen success rate of 70% matched medium-level recognition rates for MI BCI that can benefit from assistance to avoid frustration. Maintaining a fixed success rate allowed for a simpler analysis, kept the interaction time for patients to a reasonable length, and avoided fatigue. The granularity with which we controlled for success rates of reel attempts was coarse while guaranteeing a consistent number of successful reels. Consequently, we could, therefore, not control the quantity of participants’ (blink) action outcomes (for which we computed conversion rates) or task outcomes (modeled as fish caught). With another experimental design or real BCI, people might experience failed attempts followed by success within the same reel trial. In our case, once the first blink failed, people could have discovered that success was not possible during the current trial. However, none of the participants remarked on this.

Longer-term exposure to explicit help might yield experiences other than during our 20-min playthroughs and will require field studies with durations commensurate with rehabilitation stays. The genre and pacing of the game might have influenced the comparison of the help mechanisms. Faster- or slower-paced games in different guises or narratives employing similar help might yield different results.

Our participants were not screened or medically examined prior to participating. Differences in ability were not recorded and excluded from our analysis. How representative or skewed our participants were, therefore, remains unclear.

Real BCI input feels different from eye-blink input since people are aware of their blinks but cannot judge having correctly carried out a motor imagery attempt. While eye tracking lets us simulate an unreliable input method, how the difference in operation to MI BIC affects our findings remains unclear. However, the short delay in recognizing a successful blink is very similar to that incurred in MI BCI attempts.

The study surfaced limitations with the experimental design, which were not observed in previous studies [48,52]. Although patients did differentiate between help, they rated their perceived control differently to non-patients [52]. We found new relevant experiential factors (e.g., pacing, help appeal, and help quantity) that we could neither discern nor control regarding their effect on perceived control and frustration. This highlighted a need for more comprehensive measurements to quantify the experience of help and its effect on control. For example, quantifying how users experience helps when it “interferes” with individual preferences (P8). Based on the qualitative results in Study 1, a follow-up study designed and evaluated twenty new measurements to provide the necessary experiential context to understand perceived control and frustration.

## 4. Follow-Up Exploration: Refining Experiential Measures of Help

In the stroke patient study, we relied on established self-report measurements of perceived control and frustration from causal learning [50] and previous HCI studies of imprecise control [25,49,52,54]. These measurements were sufficient in binary comparisons of positive/negative feedback [50,54] but fell short when used in the context of help mechanisms with changing narratives.

We saw an opportunity to improve our measurement instruments to better capture the rich experiential space of receiving help to aid in unpacking the high variation in stroke patients’ responses. Based on the qualitative feedback from stroke patients in Study 1, we created new experimental measures and ran a follow-up exploration of the self-report instruments to identify challenges and potential measurement dimensions. To avoid burdening our vulnerable target group, we relied on healthy participants to test the revised instrument. This allowed us to assess a large pool of untested measures while removing concern for higher fatigue or cognitive load in stroke patients due to increased experimental length and complexity. We expanded the measurements from two tested to 20 untested measures (12 condition-wide questions and 8 experiment-wide questions), followed by a longer interview. Many of the untested measures represent new approaches to quantifying virtual help, which, to our knowledge, had not been evaluated before and would provide additional experiential detail of help in systems employing imprecise input devices.

The follow-up exploration was conducted four months after the data collection of the stroke patient study in a safe context with university students. We recruited students with a gaming background in the hope of obtaining higher agreement in self-report measures among raters and higher sensitivity to the experience at hand before returning to validate any measures with stroke patients. Our internal exploration study took 30–35 min to complete.

### 4.1. Measurement Design

To derive the new self-report measures, we examined patient responses for opinions that represented quantifiable contrasts. The process resulted in eight identified primary measures, presented below:

1. Help Quantity: Users’ experienced quantity of help. Different help styles may have felt like receiving more or less help, e.g., P10’s “*I didn’t really notice the clamp*” (mitigated failure) or P17’s “*It was like getting a small break when she came and helped*” (input override).
2. Help Appeal: How much did users appreciate the help they received? This is evident from the contrasting patient responses, like, for example, P10’s “*It was nice that she helped*” or P8’s “*She shouldn’t interfere in the game*”.
3. Pacing: Do users experience that help affects the game’s pacing? This is inspired by P17’s “*It was like getting a small break when she came and helped*” and P6’s “*It felt like it went slower, but I still caught many fish*”. We explored whether the help types affected people’s sense of *pacing*: we did not expect particularly high psychological immersion effects from the game’s simple narrative [68], but we wanted to see whether players picked up on the variations in gameplay duration initially observed in the stroke patient study.
4. Irritation: Do users wish to distinguish frustration and irritation when rating help? This is inspired by responses to frustration like P2’s “*Fishing* [and losing fish in a game] *is not something that annoys me*” or P7’s “*It’s not something that affects me*”.
5. Attribution: Are users blaming the system or themselves for the poor performance? For example, this is evident from patients like P18’s “*I lost way more fish this time*” (emphasising themselves) and P16’s “*It won’t do it*” (emphasizing the system).
6. Self-performance: Did users consider themselves good at playing the game? This is inspired by P5’s “*It’s fun! But also a bit hard…*”.
7. Style: Did users enjoy the way the game was styled? E.g., P18’s comment: “*It feels a bit silly*”.
8. Joy: Did users enjoy playing this game? E.g., P19’s comment: “*It was pretty fun when I finally understood how to play*” or P11’s “*Repetitive*”.

We formalized study goals for all condition-wide measures and used insights from the stroke patient study to hypothesize expected relationships between self-report measures and game environment variables in the right-most column of Table 4. In addition to the new self-report measurements, participants rated their agreement to seven chosen quotes from the debrief of the stroke patients in the stroke patient study. With this design, we created more facets to capture conditional differences and give better room for exploration than general questions.

### 4.2. Participants

The study included 20 participants (5F and 15M), of which 17 were experienced video game players (age 21–60, M = 22.79, and SD = 2.76) who signed up for the study voluntarily. The participants were university students and were unfamiliar with the project beforehand.

### 4.3. Procedure

To explore how the new measurements were performed, we used an identical procedure to Study 1. We switched Study 1’s two-question questionnaire to two much more elaborate questionnaires, which are available in Supplementary Material S2. Participants filled out a questionnaire after each condition and then an additional questionnaire when all three conditions were completed.

### 4.4. Apparatus

In response to the poor performance observed in the stroke patient study, we partially redesigned how the augmented success help mechanism was activated by the system. We ensured that augmented success could also activate in cases where a fish only needed a single reel to become caught. This ensured that the positive feedback rate for augmented success could reach the same 30% target rate compared to other help mechanisms. Beyond these changes, the study followed the experimental design from Study 1.

### 4.5. Data Analysis

Data analysis was performed in R following a procedure similar to that of the stroke patient study. Exclusively for visual depiction, we normalized violin plot data points to 0–1 and jittered them by 2%, similar to violin plots in the stroke patient study. A Bartlett’s test of sphericity checked whether any of the new variables could be further reduced by factor analysis in a meaningful manner. This was not the case (*p* = 4.52), and we built and evaluated cumulative link mixed models for each condition-wise, self-reported measure listed in Table 4. For condition-wise measures, models with the highest AIC were examined to understand variable estimates. Models predicting *help quantity* and *help appeal* were built using a filtered dataset in which the reference condition was not present since they were not estimated for the reference condition as no help was present. Seven quotes from the stroke patient study were tested as experiment-wide self-report measures and analyzed using correlation analysis and visual analysis. Participant 17 was excluded from the results due to problems with blink recognition during the experiment in the augmented success condition.

### 4.6. Results

The baseline performance indicated by the reference condition provided participants with 70% successful reeling attempts and 30% failed reeling attempts, reflected in the *Pos. Feedback* column in Table 5. Participants’ blink recognition rates in our follow-up exploration were more stable and exhibited identical blink conversion rates at 26%. As a result, participants experienced less negative feedback compared to the stroke patient study, reflected in the low mean *Fish Lost* and *Fish Unreel* counts and high mean *Fish Caught* and *Fish Reel* counts in Table 5. Our examination of the control variables (condition order and gender) did not find significant models for any self-report measures in the experiment. Input override was voted as the easiest (8/19), followed by mitigated failure (7/19), while the reference condition was rated the hardest (10/19).

#### 4.6.1. Perceived Control and Frustration

Contrary to previous findings with non-stroke participants, the participants’ experience in terms of perceived control and frustration did not differ for the four types of help. Neither did perceived control and frustration vary significantly with any of the game measures. The additional positive feedback introduced by the help (*Help Rate*) did not alter how people perceived their control. No relationships were found between participants’ perceived control, their blink conversion rate, or positive feedback. People rated perceived control and frustration inconsistently; an intraclass correlation analysis showed poor agreement between participants for both measures (perceived control ICC3 = 0.04 and frustration ICC3 = 0.06). The participants’ perceived control was highest for mitigated failure (5.37/7) and lowest for input override (4.63/7), but these differences were insignificant, with less than one on the seven-point Likert scale. Frustration ratings insignificantly differed with less than one between conditions on the seven-point Likert scale, with mitigated failure scored-lowest (2.21/7) and the reference condition highest (2.79/7).

#### 4.6.2. Help Quantity and Help Appeal

Help type significantly predicted help quantity and help appeal, which also had a significant relationship with multiple game measures listed in Table 6. Models of fish unreel, help type, and fish reel had similar AIC (180–183) and individually explained most of the variance when predicting help quantity. The significant game measures in Table 6 could not be modeled in combination, suggesting high model overlap between the game measures. Unexpectedly, the help rate did not predict the help quantity, but the help rate also varied much less between conditions than in the stroke patient study. Participants rated help quantity as the highest for input override (6.05/7) and lowest for augmented success (3.95/7). Participants caught the same number of fish on average in input override and augmented success conditions but received lower overall positive feedback in augmented success (70%) compared to input override (99%). This difference correctly reflects how augmented success by design only helps players when they are successful. Help quantity had the highest agreement among raters in this study (ICC3 = 0.42), although poor (less than 0.5) [69]. Participants found augmented success most appealing (mean help appeal = 4.63/7) and input override least (mean help appeal = 3.26/7). In further examination of the *Condition* model that compared PAMs, significant likelihood tests estimated that input override negatively affected help appeal ratings (Estimate = −1.87 and *p* = 0.003) compared to augmented success. The appeal of mitigated failure did not differ significantly from augmented success or input override.

#### 4.6.3. Pacing and Irritation

**Figure 5:**
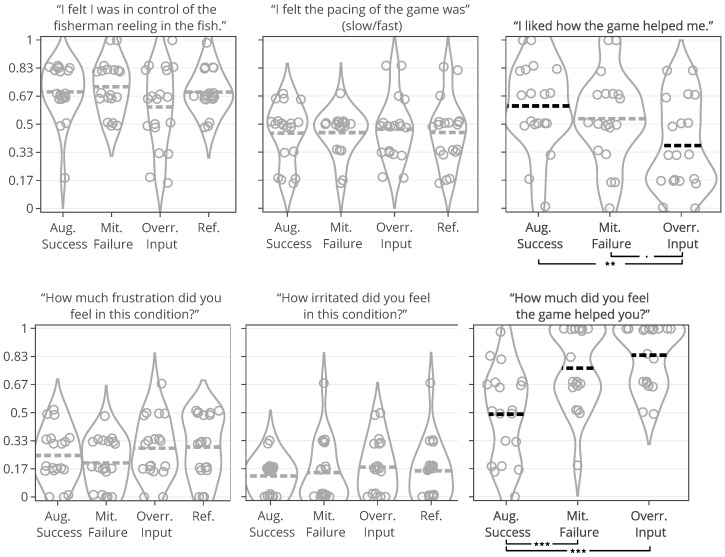
Violin plots depicting participants’ normalized condition ratings (circles) with 2% jitter and the mean (dashed line) of perceived control (**upper-left**), frustration (**lower-left**), pacing (**upper-middle**), irritation (**lower-middle**), help appeal (**upper-right**), and help quantity (**lower-right**). Stars (** and ***) and black highlight indicate significant differences.

The help type did not significantly predict pacing or irritation, and no model could be constructed, which was significantly different from the null model. Participants did not rate the pacing based on the game duration, but game duration also varied considerably less between conditions than in the stroke patient study (less than 10 s). Irritation had an observable lower spread in ratings than frustration, as shown in Figure 5. Both measures had poor ICC3 estimated at or close to 0.00.

#### 4.6.4. Agreement with Patient Viewpoints

After each help type, we asked participants to rate their agreement with the patients’ viewpoints of the help V1–V6, listed in Table 7, and performed a Spearman correlation analysis. For augmented success, the participants favored a positive viewpoint (V1), where the majority rated it five or higher, which moderately correlated with ratings of help appeal (r = 0.4). The positive viewpoint of increased success (V1) was negatively correlated (r = −0.6) with the negative view (V2) at a moderate level. Both input override viewpoints (V3 and V4) had a mixed reception, with the highest spread in participant responses to positive and negative viewpoints, and the viewpoints had a moderately reverse correlation (r = −0.6). The positive viewpoint (V3) of input override correlated moderately to participants’ ratings of help appeal (r = 0.5). The negative viewpoint (V4) correlated moderately with ratings of irritation (r = 0.4) but only weakly to ratings of frustration (r = 0.1). All participants scored mitigated failure’s positive viewpoint five or higher (V5), and most disagreed with mitigated failure’s negative viewpoint (V6), which moderately correlated with feeling good at playing this game (r = −0.5) (E6, Table 8), while the positive viewpoint (V5) moderately correlated with system-blame irritation (E2, Table 8). Positive and negative viewpoints of mitigated failure had no correlation (r = 0.0), suggesting the viewpoints were not the reverse of each other but orthogonal concepts.

#### 4.6.5. Experiment-Wide Measurements

The Spearman correlation analysis of experiment-wide variables E1–E7 listed in Table 8 showed no strong (>0.75) correlations with any self-reported ratings. Ratings of enjoying the game (E5, mean = 4.85) correlated moderately to (1) enjoying how the game was styled (E5, r = 0.5, mean = 6.25) and (2) negatively to system-blamed irritation (E2, r = −0.4). People who rated game enjoyment (E7) high did so also for perceived control (r = 0.4) and pacing (r = 0.4). People who rated enjoyment thought more about how to catch more fish (E4) (r = 0.5). Participants who did not feel irritation when losing fish (E3) tended to rate pacing higher (r = 0.5). We tested whether participants blamed the game for not registering their blinks (E2) or blamed themselves for their performance (E1) but found only a weak correlation with how participants rated perceived control (E1 r = 0.2 and E2 r = −0.2). The full correlation analysis is available as Supplementary Materials (see Supplementary Table S1).

### 4.7. Interview Analysis

Participants expressed different ideas about how the help mechanisms worked and the relationship between the blinks they performed and the success of the game. One participant believed timing the blink right influenced the outcome (P2). Other participants suggested factors such as seat position (P11) and how hard one blinked (P10). Five participants perceived that help mechanisms were activated at random and commented on the inability to decide when to receive help. “*I didn’t control when he got strong. It was a bit random. It gave me an irritated feeling*” (P19, AS). Only three participants generally commented on how the mechanisms affected the general pacing of the game. P12 described mitigating failure as “*slow*”, P14 described input override as slow, and P7 described augmented success as fast. Five of the participants (P7, P8, P11, P14, and P15) did not appreciate the game when negative feedback was completely absent. “*If the fish can’t escape, then it’s not that fun. It’s a bit frustrating*” (P15). “*Too much help. I don’t like to get babied by games*” (P12).

#### 4.7.1. Mitigated Failure

Eight participants (P8, P10, P19, P3, P4, P5, P12, and P18) found mitigated failure more appealing than other help mechanisms.“[It] saved me from making mistakes” (P10). “*It was nice when the fish stood still. It was like the clamp had control over the rod*” (P4). “*The clamp saved me, so nothing bad happened*” (P5). However, to four participants (P14, P15, P11, and P8), adding 30% mitigated failure to their 70% control meant that it removed the possibility of failing, which they disliked. “*The clamp sort of removes the consequences for me*” (P14). “*It was too easy. I just won*” (P11).

#### 4.7.2. Augmented Success

Ten participants (P3, P4, P5, P6, P14, P15, P16, and P20) felt positive towards augmented success because they felt rewarded when they blinked and augmented success reeled the fish double the distance. “*It was more rewarding. It was a better feeling when I made a good blink at the right time*” (P4). “*This was way better than the girl. It sort of amplifies what I’m already doing* [stronger reels]” (P5). The redesign of augmented success only manipulated 30% of players’ 70% successful outcomes and did not prevent people’s failures in the other 30%. Three participants (P6, P11, and P14) appreciated the design as it maintained a sense of challenge. “*Good! The fish has some liberty. Better when there is a bit of a struggle*” (P11). However, the retained failure rate meant some participants felt less in control (P13, P17, P18, and P20) and that the difficulty of the game was greater than in other conditions (P11, P12, and P13). “*I like feeling fully in control, which I don’t think I was here*” (P13). “*I felt it was equally hard to control as the first one* [The reference condition]” (P12).

#### 4.7.3. Input Override

The majority of participants (13/20) disliked the input override mechanism. Five participants (P9, P5, P6, P20, P14, and P13) directly expressed their dislike for the type of help it provided. “*Dude! I had it, okay …*” (P9). “*This also made the game really slow, because I just sat there while she fished*” (P14). Seven participants (P10, P7, P12, P13, P17, P19, and P3) disliked the quantity of help they felt the mechanism provided. “*I lose the feeling of self-accomplishment when she helps so much*” (P10). “*It was fine she helped in the beginning, but in the end it felt like she just did the work for me. I lost control from that*” (P19). The participants described feeling frustrated, feeling less in control, feeling redundant, and losing the “*competitive feeling*”. Five participants (P3, P11, P12, P13, and P16) expressed appreciation towards the input override mechanism. These participants described the help type as “*more relaxing*”, “*easier*”, and “*nice that she helped*”.

## 5. Discussion

In this paper, we evaluated how game help affected perceived control and frustration in stroke patients using a conventional experimental design and in healthy adults using an improved experimental design.

### 5.1. Study Protocol Design

The stroke patient study’s design followed the design in previous studies [48,52], which was based on the experimental design of causality studies [50]. Previous studies evaluated perceived control and frustration using BCI but had no access to ground truth, which is needed to understand the behavior of unreliable input devices. We alleviated this issue by employing blink recognition instead of a BCI, whose recognition is unknown due to a lack of access to ground truth and can vary considerably among participants. However, despite this access, we experienced a high variance in ratings of perceived control and frustration in both studies. In the stroke patient study, we attributed the variance partially to the stroke patient’s condition, the variation in blink recognition and the complexity of the game as an experience. The revised setup with healthy subjects in our follow-up exploration achieved uniform blink recognition rates at around 100% at all times, and all participants except Participant 17 completed conditions without technical difficulties during the study. However, despite this, perceived control ratings varied substantially, and we were unable to find significant predictors for them. The fishing game setting came with more experiential complexities than the settings used in the causality literature [50], and previous studies of BCI help [48]. At this point, these experiential complexities make consistent quantification of self-reported perceived control and frustration harder when the conventional experimental design is used, which is an ongoing challenge for game research [65]. In our follow-up exploration, we therefore introduced new separate constructs to obtain user perceptions of help, quantified separately from perceived control and frustration.

### 5.2. New Self-Report Measures

Perceived help quantity and help appeal were both significant differentiators between help types, which helped in understanding participants’ ratings in our follow-up study. Ratings of help quantity and help appeal both held relationships to similar game measures while only having a weak correlation (r = −0.1) with one another. For example, input override demonstrated how help quantity could be rated high but with low appeal 4.21/7. Perceived help quantity and positive feedback did not necessarily correlate. For example, augmented success, which provided 70% positive feedback, only scored 3.95/7 in perceived help quantity, whereas mitigated failure, while providing the same amount of positive feedback, appeared to provide more help 5.58/7. Perceived help quantity also did not directly correspond to sub-measures of positive feedback (number of fish reeled or caught). For example, mitigated failure only yielded 5.16 caught fish on average, whereas augmented success yielded 7.4 caught fish, but people felt they were receiving less help. These examples demonstrate how even when virtual help is introduced with a fixed rate of 30%, the help mechanisms have different effects on the task, i.e., how many fish players catch and lose. For example, augmented success and input override let players catch more fish than mitigated failure or the unassisted reference condition. On the other hand, players were more prone to losing fish in the augmented success and reference condition than in mitigated failure and input override. If people rated help mechanisms based on how many fish they caught, people would have felt most helped in augmented success and input override and found most appealing, but this was not always the case. Interviews shed light on participants’ dislike of receiving “*a lot of help*” as a cause for rating input override highest in terms of perceived help quantity but lowest in terms of appeal. Users were not confused by receiving explicit help in our studies, as observed when exposing users to partial automation or disguised help [45,48]. However, participants in our follow-up exploration sought more agency over the help mechanisms. Some described that the help happened randomly and reduced their control of what happened as a result, a phenomenon theoretically described as a *loss of control* when expecting external agency [26,27].

**Figure 6:**
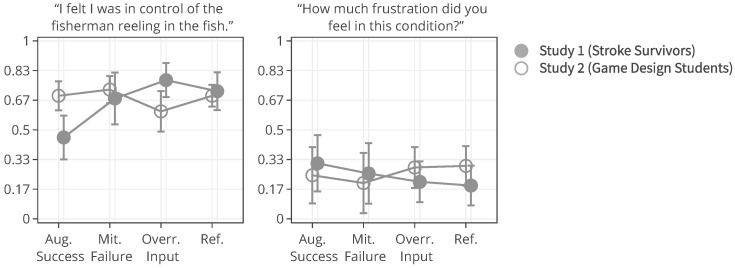
Comparison plots of mean perceived control (**left**) and frustration ratings (**right**) across three types of help by studies, with error bars indicating 95% confidence intervals.

Figure 6 shows how ratings of perceived control and frustration differed between Study 1 and Study 2, which are mostly similar except for ratings of perceived control when users received help from augmented success and override input. The protocol redesign in our follow-up exploration was created to better capture differences between conditions in a safe environment with higher control, but ratings of perceived control and frustration performed worse than recorded in the stroke patient study. Perceived control and frustration were no longer predicted by positive feedback or help type like they were in Study 1.

### 5.3. Clinical Implications of Findings

Clinicians looking to adopt BCI for stroke rehabilitation can potentially enhance patients’ training experience and long-term adherence by utilizing rehabilitation games that feature game help. The results of our studies, which simulated BCI interactions, suggest that patients’ sense of agency may increase when they receive positive feedback during BCI rehabilitation. Providing stroke survivors with tailored assistance can help personalize the rehabilitation, for example, by letting them influence how and when to receive help during training. Our proposed self-report measures of help appeal and help quantity can guide the development of BCI systems, eventually improving long-term participation in rehabilitation.

## 6. Conclusions

Digital game-based rehabilitation can be designed with help mechanisms (performance accommodation mechanisms), which shield players from frustration caused by technological limitations of imprecise input devices that reduce their sense of control. Across two studies, we evaluated how three types of games help affected users’ frustration and perceived control after playing a fishing game controlled by imprecise blink recognition. Study 1 demonstrated that perceived control depended most on the amount of positive feedback from successful and helped actions (reeled up fish) and tasks (caught fish). Patients held different opinions about the types of help. While some patients appreciated the help that removed negative feedback through failure mitigation or overriding input, other patients valued success augmentation which retained ownership of reeling in fish by themselves. The follow-up exploration with 19 healthy subjects shed light on how user perception of help can be quantified by distinguishing between the appeal of help and the perceived amount of help received. Although participants received the same percentage of help with all help mechanisms at the action level, participants subjectively perceived higher and lower quantities of help with some help mechanisms, regardless of task outcome. Experiencing more help does not always increase its appeal or lower participants’ frustration.

### Future Work

Combined, the studies have showcased the potential value of perceived control and frustration and new self-report measures for experiential evaluation of game help. Future work should further revise the proposed help appeal and help quantity measures, and eventually test them with stroke patients and explore the therapists’ role in controlling help activation. Other studies have suggested that player experience improved when players were given control over difficulty adjustment [67], based on player performance [70] or based on players’ emotions [71] (e.g., players’ self-reported emotional states through integrated dialogues with non-player characters). Further work is needed to understand how subtask-level game help tie to objective and subjective game difficulty dimensions [72,73]. Long term, we envision that the measures help quantity and help appeal could extend existing scales, such as the Gameplay Interaction Scale and its *perceived challenge* measurement construct [74], including emotional challenge (narrative choice), physical challenge (accuracy and speed of action), and cognitive challenge (reasoning, memorisation, decision-making, comprehension, and planning). With this work, we hope to pave the way for better interaction design of input assistance in games when controlled through unreliable input. With well-designed input assistance, users can perform BCI training that avoids frustration from unreliable input. In the long term, input assistance may increase motivation and active participation in repetitive motor deficit rehabilitation and, in turn, the effectiveness of therapy [32].

## Figures and Tables

**Table 1:** Explained self-report, game, and qualitative measures from the stroke patient study.

Self-Report Measures	Study Goal	Question
Perceived control (7-point scale)	Whether help types affected people’s perceived control.	“I felt I was in control of the fisherman reeling in the fish.” [52] (highly disagree→highly agree)
Frustration (7-point scale)	Whether help types affected people’s frustration.	“How much frustration did you feel in this condition?” [52] (not at all→very much)
Game Measures	Description	
Blink Conv. Rate	The percentage of blinks that resulted in reeling up a fish compared to the total number of blinks across all trials. Participants performed one or more blinks during each trial until either successful or until the black cursor had left the green area.	
Blink Recognition	Blink sensor reliability in % was computed by determining whether each trial contained an occurrence of at least one real blink (superfluous blinks were discarded in negative feedback situations). Blink recognition less than 100% indicates sensor reliability issues or that participants were not blinking as instructed in one or more trials.	
Positive Feedback	% of fish reeling attempts that resulted in positive feedback (reeling/catching a fish as a result of blinking or help).	
Help Rate	% of fish reeling attempts, which resulted in help feedback (augmented success, input override, mitigated failure).	
Fish Caught	A count of fish successfully reeled all the way up to the fisherman.	
Fish Reel/Unreel	A count of fish reeled up or down one level.	
Fish Lost	A count of fish which managed to escape (requires three unreels).	
Duration	The duration of playing the game once.	
Qualitative Measures	Description	
Video	Screen recording combined with webcam recording of players. Used by the experimenters to monitor irregularities with gameplay during post-analysis, e.g., if players didn’t blink as instructed.	
Interview Audio	Audio recording of a post-study interview with the participant. Participant responses were analyzed thematically and grouped.	

**Table 2:** Averages of participants’ absolute Likert item responses and game performance measurements for each help type and the reference condition. Standard deviation is shown in parenthesis. The ICC3 column show estimated average rating reliability for each self-report measure.

Variables	Aug. Success	Input Override	Mit. Failure	Ref. Condition	ICC3
Self-report Measures					
Perc. Control	3.75 (1.39)	5.69 (1.08)	5.07 (1.58)	5.31 (1.20)	0.36
Frustration	2.88 (1.78)	2.25 (1.29)	2.53 (1.85)	2.12 (1.26)	0.03
Hardest	7	2	3	3	0.04
Easiest	1	3	1	10	0.32
Game Measures					
Blink Recognition	85% (0.25)	90% (0.15)	93% (0.17)	86% (0.24)	-
Blink Conv. Rate	18% (0.14)	29% (0.08)	26% (0.08)	38% (0.18)	-
Pos. Feedback	30% (0.17)	87% (0.12)	58% (0.18)	60% (0.15)	-
Help Feedback	17% (0.07)	31% (0.02)	31% (0.03)	0% (0.00)	-
Fish Caught	3.31 (2.21)	8.06 (1.53)	6.13 (2.07)	5.88 (2.13)	-
Fish Lost	3.94 (1.12)	0.19 (0.54)	0.27 (0.70)	1.38 (1.31)	-
Fish Reel	2.75 (1.48)	9.31 (1.62)	5.40 (2.32)	6.06 (1.73)	-
Fish Unreel	10.00 (2.34)	2.44 (1.90)	2.00 (2.45)	6.69 (1.82)	-
Duration	190 s (17 s)	205 s (24 s)	171 s (23 s)	196 s (35 s)	-

**Table 3:** Overview of tested cumulative link mixed models, ordered by the Akaike information criterion (AIC), where a lower AIC indicates lower prediction error. It lists each model’s maximum likelihood (ML) and likelihood ratio (LR). Starred (*) $\chi^{2}$ *p*-values represent significant differences compared to the null model, where participant is the only effect.

Predicted	Fixed Effect	AIC	ML	LR	$\chi^{2}$
Perc. Control	(1|Participant) + Fish Reel	193.68	−89.84	34.91	<0.001 *
	(1|Participant) + Pos. Feedback	195.57	−90.79	33.02	<0.001 *
	(1|Participant) + Fish Lost	197.88	−91.94	30.72	<0.001 *
	(1|Participant) + Fish Unreel	204.25	−95.13	24.34	<0.001 *
	(1|Participant) + Condition	209.33	−95.67	23.26	<0.001 *
	(1|Participant) + Fish Caught	209.63	−97.81	18.97	<0.001 *
	(1|Participant) + Blink Conv. Rate	219.05	−102.52	9.55	0.002 *
Frustration	(1|Participant) + Pos. Feedback	185.12	−85.56	7.96	0.005 *
	(1|Participant) + Fish Caught	186.03	−86.01	7.05	0.008 *
	(1|Participant) + Fish Reel	186.55	−86.28	6.53	0.011 *
	(1|Participant) + Fish Lost	187.12	−86.56	5.96	0.015 *
	(1|Participant) + Blink Conv. Rate	187.26	−86.63	5.82	0.016 *
	(1|Participant) + Fish Unreel	187.72	−86.86	5.36	0.021 *

**Table 4:** List of self-report measures explored in our follow-up exploration (first column), and their study goal in relation to the help types (second column). New self-report measures introduced in our follow-up exploration are marked by an asterix (*). The third column lists the expected increasing (↑) or decreasing (↓) relationships between self-report measures and game measures.

	Study Goal	Expected Relation to Game Measures
Perceived Control	Whether help types affected people’s perceived control. (question unchanged)	↑ Increased with *Blink Conv. Rate* and *Help Rate*. ↑ Increased by no. of *Fish Caught* and *Fish Reeled*. ↓ Reduced by the no. of *Fish Lost* and *Fish Unreel*.
Frustration	Whether help types affected people’s frustration. (question unchanged)	↑ Increased by no. of *Fish Lost* and *Fish Unreel*. ↓ Reduced by *Blink Conv. Rate* and *Help Rate*.
Help Quantity *	Whether help types moderated people’s perception of how much help they received. *“How much did you feel the game helped you?”*	↑ Increased by *Help Rate*. ↓ Reduced by no. of *Fish Lost* and *Fish Unreel*.
Help Appeal *	Whether people appreciated types of help differently (for example given differences in depiction, narration and outcome). *“I like how the game helped me.”*	↑ Increased by no. of *Fish Caught* and *Fish Reeled*. ↓ Reduced by no. of *Fish Lost* and *Fish Unreel*.
Pacing *	Whether help types moderated how people felt the game in terms of speed. *“I felt like the game went fast.”*	↓ Reduced by increasing *Duration*.
Irritation *	Whether help type differ in irritation. Whether people make differentiation between frustration from irritation. *“How irritated did you feel in this condition?”*	(Same as Frustration). ↑ Increased by no. of *Fish Lost* and *Fish Unreel*. ↓ Reduced by *Blink Conv. Rate* and *Help Rate*.

**Table 5:** Average and standard deviation of participant ratings and game performance measurements for the conditions. The ICC3 column scores average rating consistency.

Self-Report Measures	Aug. Success	Input Override	Mit. Failure	Ref. Condition	ICC3
Perc. Control	5.16 (1.01)	4.63 (1.42)	5.37 (0.96)	5.16 (0.76)	0.04
Frustration	2.47 (0.96)	2.74 (1.10)	2.21 (0.92)	2.79 (1.13)	0.06
Help Quantity	3.95 (1.65)	6.05 (1.13)	5.58 (1.46)	-	0.42
Help Appeal	4.63 (1.57)	3.26 (1.59)	4.21 (1.58)	-	0.18
Pacing	3.68 (1.06)	3.84 (1.12)	3.68 (0.75)	3.68 (1.16)	0.00
Irritation	1.74 (0.65)	2.05 (1.03)	1.84 (1.12)	1.89 (1.05)	0.00
Hardest	2	4	3	10	0.14
Easiest	4	8	7	0	0.14
**Game Measures**					
Blink Recognition	99% (0.02)	99% (0.03)	98% (0.04)	99% (0.04)	-
Blink Conv. Rate	26% (0.04)	25% (0.04)	26% (0.04)	26% (0.04)	-
Pos. Feedback	70% (0.00)	99% (0.03)	69% (0.03)	70% (0.00)	-
Help Rate	28% (0.02)	31% (0.03)	30% (0.00)	0% (0.00)	-
Fish Caught	7.68 (0.67)	7.68 (0.67)	5.16 (0.50)	5.32 (0.58)	-
Fish Lost	0.21 (0.42)	0.00 (0.00)	0.00 (0.00)	0.53 (0.51)	-
Fish Reel	6.32 (0.67)	12.11 (0.81)	8.63 (0.60)	8.68 (0.58)	-
Fish Unreel	5.79 (0.42)	0.21 (0.54)	0.21 (0.54)	5.47 (0.51)	-
Duration	179 s (9 s)	180 s (8 s)	144 s (7 s)	146 s (5 s)	-

**Table 6:** Cumulative link mixed models, ordered by the Akaike information criterion (AIC), where a lower AIC means lower prediction error. ML is the maximum likelihood, and LR is the likelihood ratio. Starred (*) $\chi^{2}$ *p*-values mark significant differences to null models with the participant as the only fixed effect.

Predicted	Fixed Effect	AIC	ML	LR	$\chi^{2}$
Perc. Control	(1|Participant) + Help Appeal	233.07	−104.53	11.97	0.063
Frustration	(1|Participant) + Condition Order	219.36	−101.68	7.02	0.071
Help Quantity	(1|Participant) + Fish Unreel	179.64	−81.82	21.80	<0.001 *
	(1|Participant) + Condition	181.70	−81.85	21.73	<0.001 *
	(1|Participant) + Fish Reel	182.68	−83.34	18.76	<0.001 *
	(1|Participant) + Blink Conv. Rate	192.78	−88.39	8.66	0.003 *
	(1|Participant) + Pos. Feedback	192.94	−88.47	8.50	0.004 *
Help Appeal	(1|Participant) + Fish Reel	214.36	−99.18	9.46	0.002 *
	(1|Participant) + Pos. Feedback	216.91	−100.45	6.91	0.009 *
	(1|Participant) + Condition	217.27	−99.64	8.55	0.014 *
	(1|Participant) + Pacing	217.99	−98.00	11.83	0.019 *
	(1|Participant) + Blink Conv. Rate	219.33	−101.66	4.49	0.034 *
	(1|Participant) + Fish Unreel	219.49	−101.75	4.33	0.038 *
Irritation	(1|Participant) + Fish Reel	173.64	−80.82	1.67	0.196
Pacing	(1|Participant) + Fish Caught	179.71	−83.86	1.29	0.257

**Table 7:** Participant ratings of positive and negative stroke patient viewpoints by help type.

	Help Type	Agreement to Stroke Patient Viewpoints	Mean	SD	Spread (1–7)
V1	Aug. Success	“I think it was useful that he got strong and helped me reel in the fish.”—P9, Positive	5.11	1.70	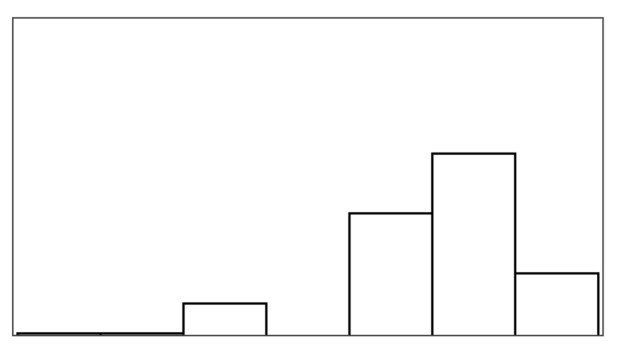
V2	Aug. Success	“He got stronger, but I didn’t think it helped me much.”—P10, Negative	3.11	1.73	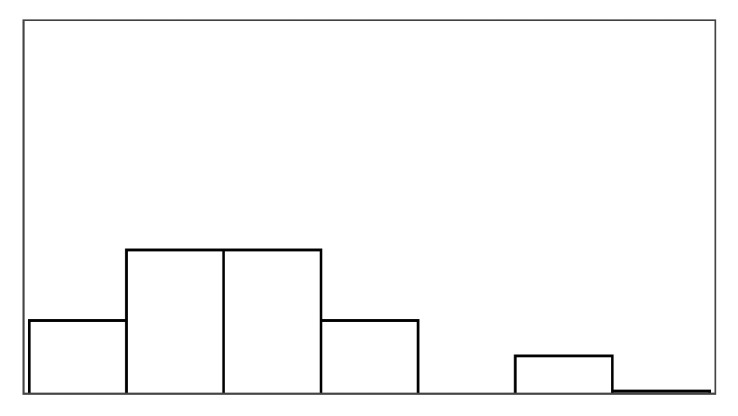
V3	Input Overr.	“I liked it when she took the fish up a notch at times, when I couldn’t”—P10, Positive	3.84	1.74	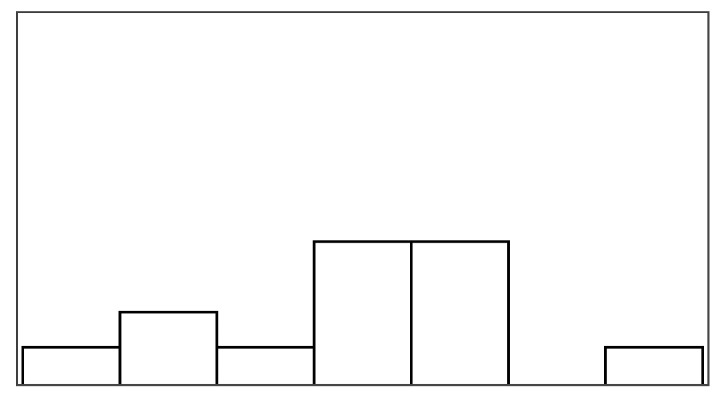
V4	Input Overr.	“It irritated me that she interfered with the game.”—P8, Negative	4.26	2.00	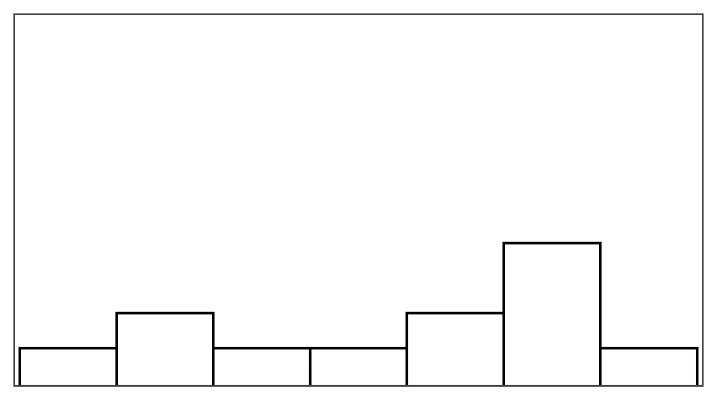
V5	Mit. Failure	“When the fish stood still, it was like saying “Let’s just try that again!”—P5, Positive	5.79	1.44	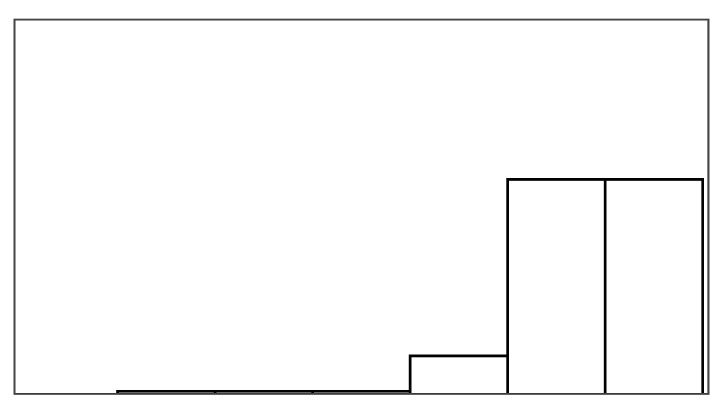
V6	Mit. Failure	“When the fish stood still, it felt like the game went slower.”—P18, Negative	3.16	1.64	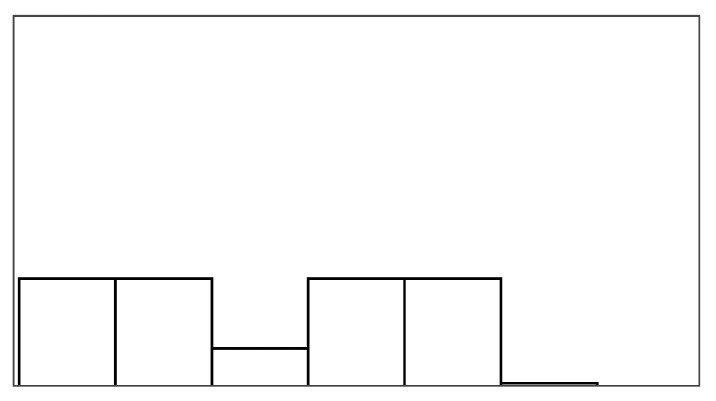

**Table 8:** Participant answers on experiment-wide questionnaire items on a seven-point scale.

	Question	Mean	SD	Spread (1–7)
E1	“It irritated me how bad I was at blinking correctly.”	3.55	2.16	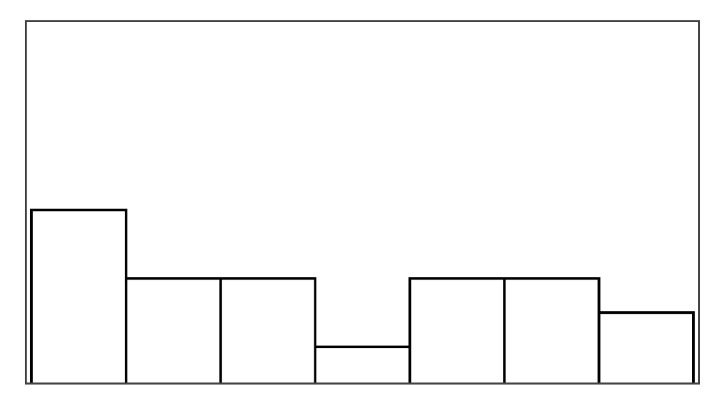
E2	“It irritated me when the game did not register my blinks.”	5.25	1.41	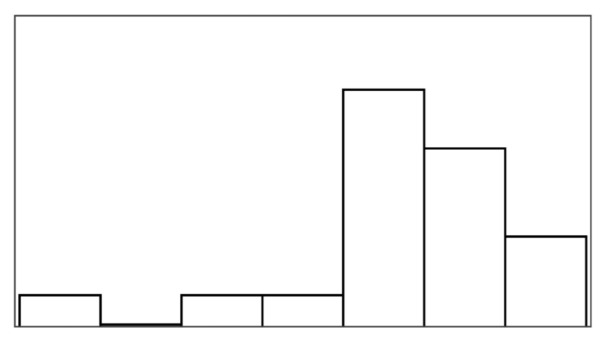
E3	“Losing the fish is not something that irritates me.”	3.65	1.81	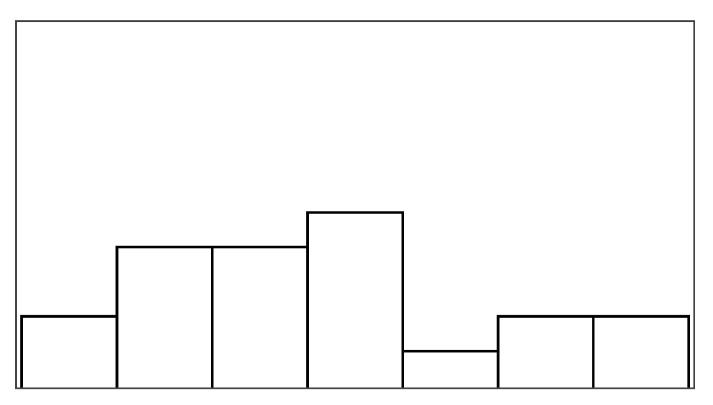
E4	“I was thinking about how I could be better at catching fish.”	4.95	1.82	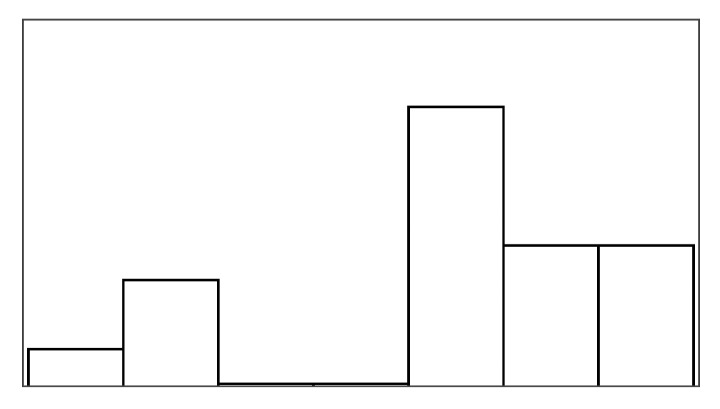
E5	“I enjoyed the way the game was styled.”	6.25	0.79	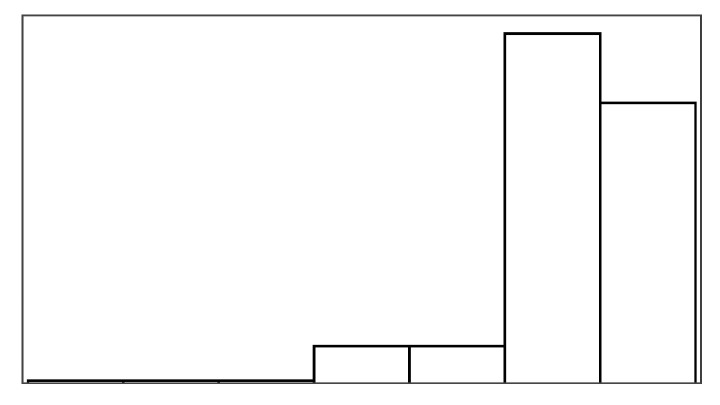
E6	“I felt I was good at playing this game.”	4.60	1.39	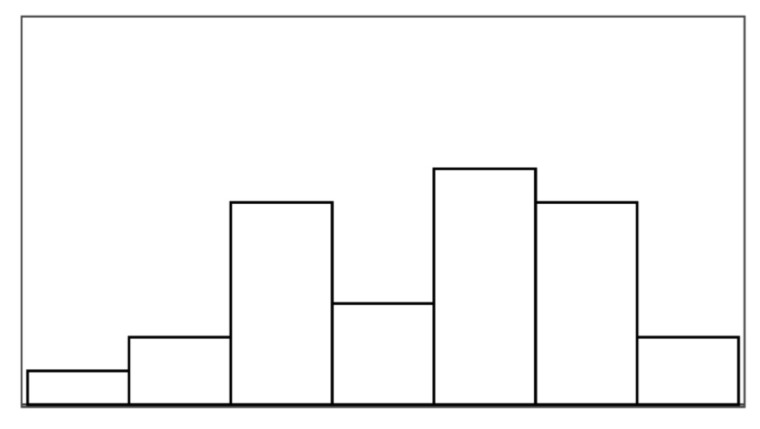
E7	“I enjoyed playing this game very much.”	4.85	1.14	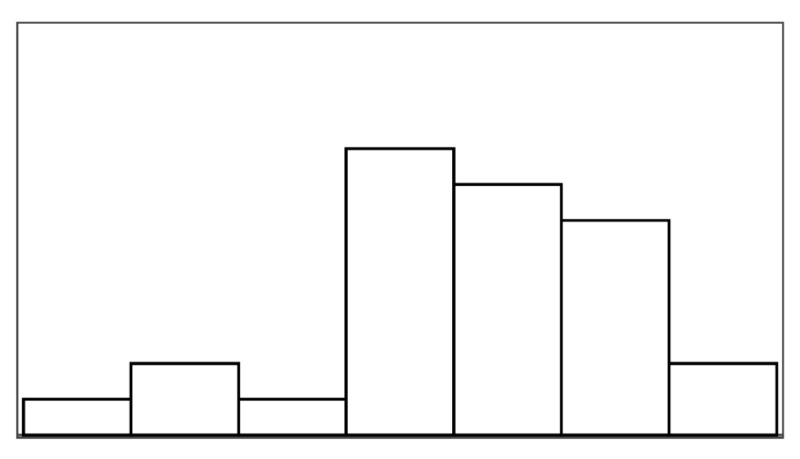

## Data Availability

The data supporting the findings of this study are available upon reasonable request from the corresponding author.

## Supplementary Materials

The following supporting information can be downloaded at https://www.mdpi.com/article/10.3390/s25092742/s1, including Supplementary Material S1, Supplementary Table S1, and Supplementary Material S2.
