# Supplementary material for: Experience of Virtual Help in a Simulated BCI Stroke Rehabilitation Serious Game and How to Measure It
Source: Sensors (Basel). 2025 Apr 26;25(9):2742. doi: 10.3390/s25092742 (PMC12074238; doi:10.3390/s25092742)
Supplement: Supplementary file 1 [file sensors-25-02742-s001.zip › supplementary_material_S1.pdf]

Supplementary Material 1

Experiment Introduction - Study 1

In the experiment, you will get to play 4 flavours of a computer game, which you control with your brain via blinking. Afterwards, you will rate your experience. We are testing different algorithms for measuring your brain signals in a game. We need you to evaluate which algorithms gave you the sense that you were in control of the game.

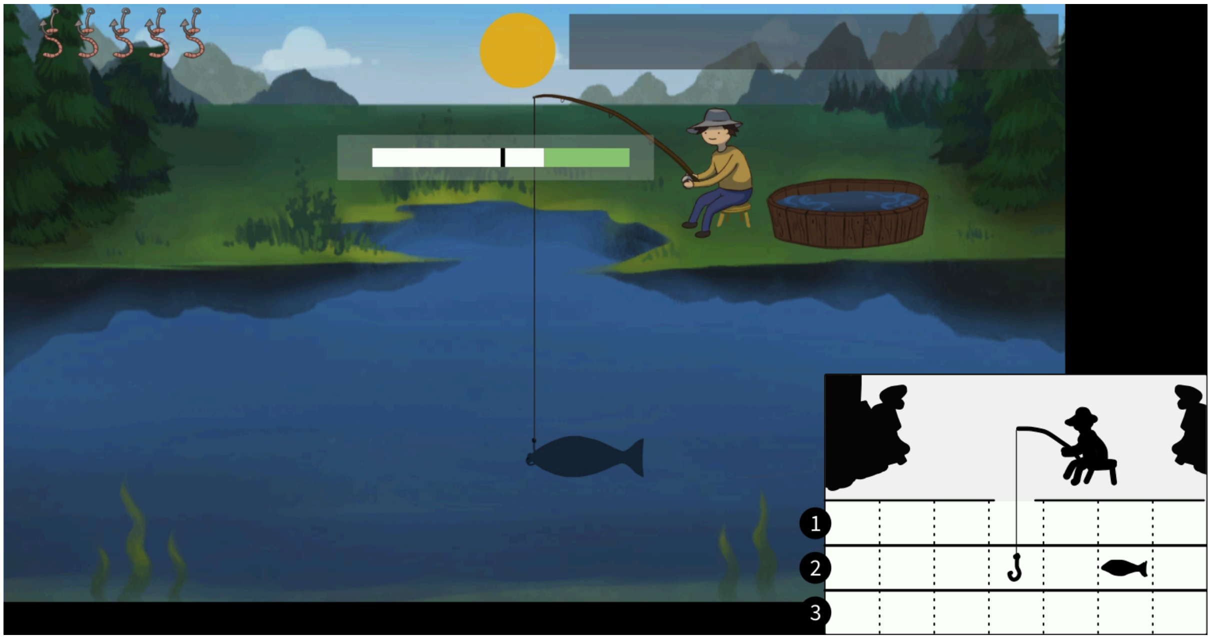

[Participants were then shown the image above on a printout. Elements in the image were explained.]

Debrief - Semi-structured Interview Guide for Study 1

[Instruction to facilitator: Go through participants mood and expectations for the experiment.]

- Now that you have tried all the conditions, what was your impression of the experiment, what do you perceive we are testing? How did you rate?
- What was your expectations prior to the experiment? How did your experience live up to the expectations?
- What previous experience with BCI do you have?
- *[Gently ask whether participants felt any potential fatigue.]*

[Instruction to facilitator: Go through why the participant rated as they did.]

- *[Point to the filled likert scale.]* What do you think about the help mechanics?
- Which help mechanic was hardest to control? Which was easiest?
- On a scale from 0-100% what do you think is the chance you could reel up the fish correctly?

[Reveal the nature of the study's conditions to the participants and how help mechanics work.]

Questionnaire for Study 1: Stroke Patients' Perceptions of Game Help

Participant No. Age Gender

Please rate your experience as a whole during this playthrough.

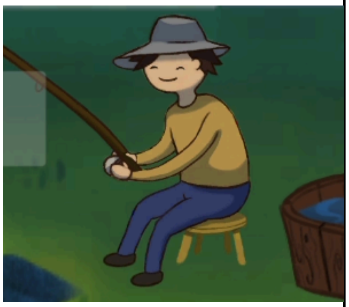

I felt I was in control of the fisherman reeling in the fish.

Strongly Disagree Strongly Agree

1 2 3 4 5 6 7

How much frustration did you feel in this condition?

Absent Strongly Pronounced

1 2 3 4 5 6 7

Please rate your experience as a whole during this playthrough.

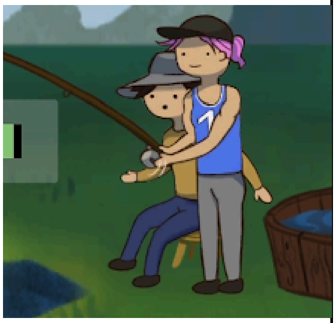

I felt I was in control of the fisherman reeling in the fish.

Strongly Disagree Strongly Agree

1 2 3 4 5 6 7

How much frustration did you feel in this condition?

Absent Strongly Pronounced

1 2 3 4 5 6 7

Please rate your experience as a whole during this playthrough.

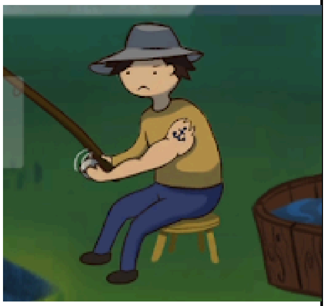

I felt I was in control of the fisherman reeling in the fish.

Strongly Disagree Strongly Agree

1 2 3 4 5 6 7

How much frustration did you feel in this condition?

Absent Strongly Pronounced

1 2 3 4 5 6 7

Please rate your experience as a whole during this playthrough.

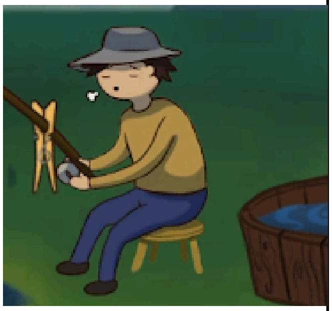

I felt I was in control of the fisherman reeling in the fish.

Strongly Disagree Strongly Agree

1 2 3 4 5 6 7

How much frustration did you feel in this condition?

Absent Strongly Pronounced

1 2 3 4 5 6 7
