# Supplementary material for: Experience of Virtual Help in a Simulated BCI Stroke Rehabilitation Serious Game and How to Measure It
Source: Sensors (Basel). 2025 Apr 26;25(9):2742. doi: 10.3390/s25092742 (PMC12074238; doi:10.3390/s25092742)
Supplement: Supplementary file 1 [file sensors-25-02742-s001.zip › supplementary_material_S2.pdf]

Study 2 Questionnaire: Exploring Methodology with Experienced Video Game Players

Participant No. Age Gender

Please rate your experience as a whole during this playthrough.

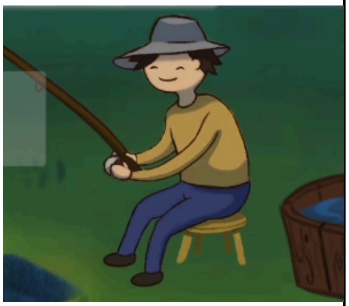

I felt I was in control of the fisherman reeling in the fish.

Strongly Disagree ... Strongly Agree

1 2 3 4 5 6 7

How much frustration did you feel in this condition?

Absent ... Strongly Pronounced

1 2 3 4 5 6 7

I felt the pacing of the game was

Too slow ... Just right ... Too fast

1 2 3 4 5 6 7

How irritated did you feel in this condition?

Not at all ... A lot

1 2 3 4 5 6 7

Study 2 Questionnaire: Exploring Methodology with Experienced Video Game Players

Participant No. Age Gender

Please rate your experience as a whole during this playthrough.

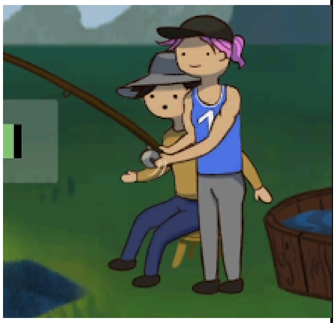

I felt I was in control of the fisherman reeling in the fish.

Strongly Disagree — Strongly Agree

1 2 3 4 5 6 7

How much frustration did you feel in this condition?

Absent — Strongly Pronounced

1 2 3 4 5 6 7

How much did you feel the game helped you?

Not at all — A lot

1 2 3 4 5 6 7

I like how the game helped me.

Strongly Disagree — Strongly Agree

1 2 3 4 5 6 7

I felt the pacing of the game was

Too slow — Just right — Too fast

1 2 3 4 5 6 7

How irritated did you feel in this condition?

Not at all — A lot

1 2 3 4 5 6 7

“I liked it when she took the fish up a notch at times, when I couldn’t”.

Strongly Disagree — Strongly Agree

1 2 3 4 5 6 7

"It irritated me that she interefered with the game."

Strongly Disagree — Strongly Agree

1 2 3 4 5 6 7

Study 2 Questionnaire: Exploring Methodology with Experienced Video Game Players

Participant No. Age Gender

Please rate your experience as a whole during this playthrough.

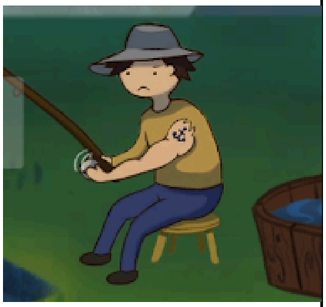

I felt I was in control of the fisherman reeling in the fish.

Strongly Disagree — Strongly Agree

1 2 3 4 5 6 7

How much frustration did you feel in this condition?

Absent — Strongly Pronounced

1 2 3 4 5 6 7

How much did you feel the game helped you?

Not at all — A lot

1 2 3 4 5 6 7

I like how the game helped me.

Strongly Disagree — Strongly Agree

1 2 3 4 5 6 7

I felt the pacing of the game was

Too slow — Just right — Too fast

1 2 3 4 5 6 7

How irritated did you feel in this condition?

Not at all — A lot

1 2 3 4 5 6 7

"I think it was useful that he got strong and helped me reel in the fish."

Strongly Disagree — Strongly Agree

1 2 3 4 5 6 7

"He got stronger, but I didn't think it helped me much."

Strongly Disagree — Strongly Agree

1 2 3 4 5 6 7

Study 2 Questionnaire: Exploring Methodology with Experienced Video Game Players

Participant No. Age Gender

Please rate your experience as a whole during this playthrough.

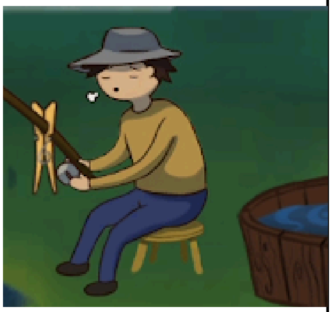

I felt I was in control of the fisherman reeling in the fish.

Strongly Disagree — Strongly Agree

1 2 3 4 5 6 7

How much frustration did you feel in this condition?

Absent — Strongly Pronounced

1 2 3 4 5 6 7

How much did you feel the game helped you?

Not at all — A lot

1 2 3 4 5 6 7

I like how the game helped me.

Strongly Disagree — Strongly Agree

1 2 3 4 5 6 7

I felt the pacing of the game was

Too slow — Just right — Too fast

1 2 3 4 5 6 7

How irritated did you feel in this condition?

Not at all — A lot

1 2 3 4 5 6 7

“When the fish stood still, it was like saying “Let’s just try that again!”

Strongly Disagree — Strongly Agree

1 2 3 4 5 6 7

"When the fish stood still, it felt like the game went slower."

Strongly Disagree — Strongly Agree

1 2 3 4 5 6 7

Study 2 Questionnaire: Exploring Methodology with Experienced Video Game Players

Participant No. Age Gender

Post-experiment questionnaire

**What adjectives describes best the sensation when the game did not pick up what you were trying to do?**	
**Dansk**<br>☐ Træls<br>☐ Irriterende<br>☐ Urimelig<br>☐ Pjattet<br>☐ Besværlig<br>☐ “Jeg sukkede meget da jeg spillede”<br>☐ ____________________	**English**<br>☐ Annoying<br>☐ Challenged<br>☐ Unfair<br>☐ Irritating<br>☐ Disturbing<br>☐ “I sighed a lot when I played”<br>☐ ____________

"It irritated me when the game did not register my blinks."

Strongly Disagree Strongly Agree

1 2 3 4 5 6 7

"It irritated me how bad I was at blinking correctly"

Strongly Disagree Strongly Agree

1 2 3 4 5 6 7

What did you think about most when you rated your control of the fisherman reeling the fish?

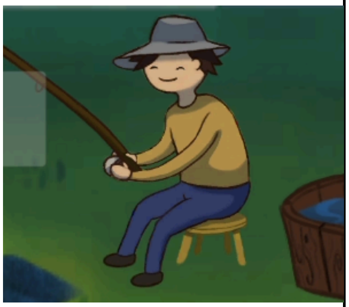

..How much I had to blink

Strongly Disagree Strongly Agree

1 2 3 4 5 6 7

..How much I managed to reel the fish up.

Strongly Disagree Strongly Agree

1 2 3 4 5 6 7

..How many fish I managed to catch

Strongly Disagree Strongly Agree

1 2 3 4 5 6 7

..How many fish I lost

Strongly Disagree Strongly Agree

1 2 3 4 5 6 7
